# Cytotoxicity and Oxidative Stress Responses of Imidacloprid and Glyphosate in Human Prostate Epithelial WPM-Y.1 Cell Line

**DOI:** 10.1155/2020/4364650

**Published:** 2020-12-08

**Authors:** Khaled Y. Abdel-Halim, Safaa R. Osman

**Affiliations:** Mammalian & Aquatic Toxicology Department, Central Agricultural Pesticides Laboratory, Agricultural Research Center (ARC), 12618-Dokki, Giza, Egypt

## Abstract

Insecticide imidacloprid and herbicide glyphosate have a broad spectrum of applicable use in the agricultural sector of Egypt. Their ability to induce in vitro cytotoxic and oxidative stress on normal human cells (prostate epithelial WPM-Y.1 cell line) was evaluated with the methyl tetrazolium test (MTT) and histopathological investigation. Cell viability was evaluated with an MTT test for 24 h. The median inhibition concentration (IC_50_) values were 0.023 and 0.025 mM for imidacloprid and glyphosate, respectively. Sublethal concentrations: 1/10 and 1/50 of IC_50_ and IC_50_ levels significantly induced an increase in the lactate dehydrogenase (LDH) activity and malondialdehyde (MDA) level compared with the untreated cells. Rapid decrease in the glutathione (GSH) content and glutathione-S-transferase (GST) activity was induced. Significant increases were recorded in activities of catalase (CAT), glutathione peroxidase (GPx), and glutathione reductase (GR), respectively, compared with the control group. Transmission electron microscopic (TEM) investigation showed significant defects in the cells following pesticide treatments for 24 h. Therefore, it is concluded that imidacloprid and glyphosate are very toxic in vitro assays and able to induce apoptotic effects as well as oxidative stress. So, these findings provide a scenario of multibiomarkers to achieve the imposed risks of pesticides at low doses.

## 1. Introduction

Insecticide imidacloprid (1-(6-chloro-3-pyridylmethyl)-*N*-nitroimidazolidin-2-ylideneamine; CAS number 138261-41-3) is used to control sucking and some chewing insects. It can be topically applied to pets, as well as to structures, crops, soil, and as a seed treatment, to control fleas [1]. It is used to protect seedlings from the early season root and leaf feeding pests, as well as in later season foliar treatments [2]. On the contrary, glyphosate (*N*-(phosphonomethyl)glycine; CAS number 1071-83-6) is a broad-spectrum systemic herbicide and crop desiccant. It is used to kill weeds, especially annual broadleaf weeds and grasses that compete with crops. In 2007, glyphosate was the most used herbicide in the United States, agricultural sector, and the second-most used in gardens, industry, and commerce. Since 2016, there was 100-fold increase for the late 1970s in the frequency of application and volume of glyphosate-based herbicides (GBHs) applied, with further increases expected in the future partly in response to the global emergence and spread of glyphosate-resistant weed [3]. In Egypt, it is extensively used to control weeds in beans, grapes, and citrus crops and to control annual and biannual weeds.

In recent years, correlative studies have indicated that pesticides routinely used in crop protection may have detrimental effects upon human health. As the result of the widespread use and the lack of safe management of pesticides in developing countries, various compartments of the environment are contaminated and exposure to pesticides is a concern toward the general population [4, 5]. For example, neonicotinoid insecticides are derivates of nicotine and classified as *N*-nitroguanidines (imidacloprid, thiamethoxam, dinotefuran, and clothianidin) and *N*-cyano-amides (acetamiprid and thiacloprid). They have broad spectrum practices as insecticides in the agricultural sector and are effective at low dosage and have poor affinity for the nicotinic acetylcholine receptor in mammalian species [6, 7]. They are potent selective agonists of the nicotine acetylcholine receptor (nAChR) in both invertebrates and vertebrates [6, 8, 9]. They are classified by EPA as class II and III toxins and labeled with a signal word “warning” or “caution” [10–12]. There are reports of neonicotinoid poisoning in humans and others. Imidacloprid was primarily negative in vitro gene mutation assays in bacterial and mammalian cells. However, positive results were seen in vitro chromosome aberration and sister chromatid exchange assays, mostly at cytotoxic doses [13]. Also, it mediated CYP19 expression and aromatase catalytic activity in human umbilical vein endothelial cells (HUVEC) [14]. As stated in the literature, imidacloprid caused DNA strand breaks in the coelomocytes of earthworm, *Eisenia fetida* [15], tadpole erythrocytes from Rana N-Hallowell [16], human peripheral blood lymphocytes [17, 18], and leukocytes in culture [19]. It is important to evaluate the cytotoxic actions of the pesticides to contribute with toxicological data and regular use without polluting the environment and safe food and drinks.

On the contrary, glyphosate and glyphosate-based herbicides are major pollutants of rivers, surface water, and food [20]. Several studies have shown that glyphosate reveals adverse effects to humans including endocrine disrupting activity [21, 22]. Also, it has been used for over 40 years, and the assessment of toxic potential still demands significant verification. Various studies demonstrated that it was cytotoxic at high concentrations [23, 24]. These potential side effects are due to glyphosate's extensive agricultural use worldwide. In in vitro, some studies have yielded inconsistent results regarding glyphosate's cytotoxic properties. A study was conducted by Gasnier et al. [23] showed that toxicity on HepG2 cells appearing at glyphosate concentrations is less than 5 ppm during the incubation period (24 h), but concentrations of 120 nM induced DNA damage after the same period. However, concentrations of 15, 25, and 50 mM of glyphosate did not decrease cell viability in epithelial cell lines RWEP-1 and pRNA-1-1 and in normal cells [25]. Recently, the World Health Organization (WHO), in March 2015, decided the classification of glyphosate on category 2A, as probable carcinogenic to humans [26].

In in vitro studies, cell viability and cytotoxicity assays on cultured cells are widely used for tests of chemicals and for drug screening. Application of these assays has increasing uses over recent years. Cell viability and cytotoxicity assays are based on various cell functions such as cell membrane permeability, enzyme activity, cell adherence, ATP production, coenzyme production, and nucleotide uptake activity [27]. Moreover, pesticides are individually known to induce toxicity at the cellular level throughout oxidant-mediated responses such as apoptotic or necrotic cell death, membrane lipid peroxidation (LPO), metabolic perturbation, deregulation of several signaling pathways [28], or alteration of tight junctions [29, 30]. As above, the study aims to evaluate the potential cytotoxic effects and oxidative stress induction of imidacloprid and glyphosate on WPM-Y.1 cell line in coupling with histopathological alterations profiles following 24 h exposure.

## 2. Materials and Methods

### 2.1. Chemicals

Technical (purity, 95%) of glyphosate (*N*-(phosphonomethyl) glycine; CAS number 1071-83-6) and imidacloprid (*N*-(1-((6-chloro-3-pyridyl)methyl)-4,5-dihydroimidazol-2-yl-nitramide); CAS number 138261-41-3) were purchased from Kafr El-Zayat Company for Pesticides & Fertilizers, Egypt.

Reagents 3-(4,5-dimethylthiazol-2-yl)-2,5-diphenyltetrazolium bromide (MTT), glutaraldehyde, sodium pyruvate, thiobarbituric acid (TBA), 5,5′-dithiobis 2-nitrobenzoic acid (DTNB), 1-chloro-2,4-dinitrobenzene (CDNB), reduced glutathione (GSH), oxidized glutathione (GSSG), *β*-nicotinamide adenine dinucleotide reduced form (*β*-NADPH), bovine serum albumin (BSA), osmium tetraoxide (OSO_4_), propylene oxide, epon araldite, and toluidine blue were obtained from Sigma Chemical Co., P.B. 14508S2, Louis MO 63178, USA. Solvents: isopropanol and acetone and hydrochloric acid (HCl), salts: potassium phosphate mono and dibase, ethylene diamine tetraacetic acid (EDTA), trichloroacetic acid (TCA), hydrogen peroxide (H_2_O_2_), uranyl acetate, and lead acetate were obtained from BDH Chemicals Ltd. Pool, England. All reagents were prepared in deionized water and adjusted for pH values as required.

### 2.2. Cell Culture

Prostate human cell line WPM-Y.1 was provided by Medical Technology Center (MTC), Medical Research Institution, Alexandria University, Egypt. The cells were maintained in a standard medium consisting of DEMEM with 10% (v/v) fetal bovine serum and 1% (v/v) penicillin/streptomycin and incubated at 37°C in a 5% CO_2_ prior to use. The medium was replaced with fresh DEMEM-10% in phosphate-buffered saline (PBS). The cells were maintained by subculturing them after arriving at an acceptable confluence.

### 2.3. Toxicity Test

The cells were seeded into 96-well cell culture plates at a concentration of 1 × 10^4^ cells ml^−1^ and incubated for 24 h at standard conditions to reach exponential growth. The cells were treated with different concentrations of the examined pesticides ranged from 0.025 to 0.4 *μ*g·ml^−1^. After 24 h of incubation, the medium was removed and 5 mg·ml^−1^ of MTT reagent was added to each plate and left to incubate for 3-4 h. The formazan crystals were dissolved in 100 *μ*l acidified isopropanol and read at 630 nm by using an ELISA microplate reader (Bio-RAD microplate reader, Japan). Each concentration was repeated in triplicates. The fields of untreated and pesticide-treated cells were visualized on light microscope to compare the defects on the cells after the exposure period. Cell viability was calculated as follows:

(1) $$\begin{array}{c} \text{cell viability }\%=\frac{\mathrm{AbsS}\;\times \;100}{\text{Abs }C}, \end{array}$$

where Abs S and Abs C were absorbance of the cells incubated with samples and without sample, respectively.

### 2.4. Sublethal Acute Toxicity

Two levels, 1/10 and 1/50 IC_50_, for each compound were used. For each concentration, 3 replicates were used in a T-25 culture flask as described above. After 24 h of incubation, the medium was removed and the remaining cells were harvested in PBS and centrifuged at 2000 rpm for 10 min at 4°C. For enzyme assays, the media and cell lysate were stored at −80°C until used. However, an aliquot (5 ml) of suspension was taken for ultrastructural investigation, where it was centrifuged at 3000 rpm for 5 min, the supernatant was discarded, the pellet was suspended in 2 ml of 2.5% glutaraldehyde (0.1 M phosphate buffer pH 7.2), and stored at 4°C until used.

### 2.5. Biochemical Quantifications

#### 2.5.1. Cell Lysate

The pellets were homogenized in cold (50 mM potassium phosphate buffer pH 7.5, 2.0 mM EDTA). The homogenate was centrifuged at 4000 rpm for 15 min at 4°C. The cell lysis was used as a source for lactate dehydrogenase (LDH), LPO, and glutathione (GSH) content assays, but the supernatant was used for catalase (CAT), glutathione-S-transferase (GST), glutathione reductase (GR), and glutathione peroxidase (GPx) assays.

#### 2.5.2. LDH

The enzyme activity was measured by the method of Mc Queen [31] by using sodium pyruvate as a substrate. The activity was expressed as U·L^−1^.

#### 2.5.3. LPO

The thiobarbituric acid reactive substances (TBARS) were used as an index of LPO according to Rice-Evans et al. [32] with modification. TBARS was determined by spectrophotometric quantification of the malondialdehyde (MDA) content. An aliquot (250 *μ*l) of cell lysate or media was mixed with 1 ml of 15% (w/v) trichloroacetic acid (TCA) in 25 mM HCl and 2 ml of 0.37% (w/v) thiobarbituric acid (TBA). The mixture was boiled for 10 min, quickly cooled, and immediately centrifuged at 5000 rpm for 5 min. The absorbance was determined at 535 nm. MDA was quantified using an extinction coefficient of 156 mM^−1^, and its concentration was expressed as mM·g^−1^ tissue.

#### 2.5.4. GSH

The method was based on the reduction of 5,5′-dithiobis 2-nitrobenzoic acid (DTNB) with GSH to produce a yellow compound measured at 405 nm [33]. To 500 *μ*l of enzyme source, the same volume of 500 mM TCA was added, followed by centrifugation at 3000 rpm for 15 min. An aliquot (500 *μ*l) was well mixed with 1 ml of each 100 mM PBS buffer, pH 7.4, and 1 mM DTNB. After 10 min, the absorbance was measured at 405 nm against the blank. The GSH concentration was expressed as nMg^−1^ tissue.

#### 2.5.5. CAT

The enzyme activity was measured following the decrease in absorbance at 240 nm due to hydrogen peroxide (H_2_O_2_) consumption [34]. The reaction mixture consisted of 1 ml of 12.5 mM H_2_O_2_ (substrate), 2 ml of 66.7 mM phosphate buffer, pH 7.0, and an aliquot of enzyme source. The activity was expressed as Ug^−1^ tissue. The unit of CAT is the amount of enzyme, which liberates half the peroxide oxygen from the H_2_O_2_ solution of any concentration in 100 *μ*l at 25°C.

#### 2.5.6. GST

The activity was determined by the spectrophotometric method of Habig and Jakoby [35] by using 1-chloro-2-4 dinitrobenzene (CDNB). Enzyme source was mixed with 500 *μ*l of potassium phosphate buffer (50 mM; pH 6.5). The incubation was done at 25°C for 5 min, followed by adding 100 *μ*l of 0.2 M CDNB and 150 *μ*l of 10 mM GSH. After 1 min, the change in absorbance was recorded every 30 s for 6 min at 340 nm. The enzyme activity was expressed as nM·mg^−1^·min^−1^.

#### 2.5.7. GPx

The enzyme activity was measured according to Flohe and Gunzler [36] by mixing phosphate buffer solution (100 mM), EDTA (50 mM), sodium azide (250 mM), H_2_O_2_ (10 mM), and enzyme in a cuvette. The change in absorbance was recorded every 3 s for 40 s at 340 nm. The activity was expressed as m·U·GPx·mg^−1^ protein. One unit of GPx is defined as the amount of enzyme necessary to oxidize 1 *μ*M of NADPH per min.

#### 2.5.8. GR

The activity of GR was measured by following the decrease in the absorbance during NADPH oxidation [37]. In each cuvette, 0.1 M potassium phosphate buffer, 3.4 mM EDTA, pH 7.6, 30 mM oxidized glutathione (GSSG), 0.8 mM *β*-NADPH, and 1.0% of bovine serum albumin (BSA) were mixed by inversion. Then, 100 *μ*l of the enzyme was added. The absorbance was recorded at 340 nm for approximately 5 min. The activity was expressed as U·mg^−1^ protein. One unit will reduce 1.0 *μ*M of GSSG per min at pH 7.6 and 25°C.

#### 2.5.9. Protein Content

Total protein was determined according to the method of Lowry et al. [38]. The intensity of the developed blue colour was measured at 750 nm against the blank. BSA was used as a standard.

### 2.6. Ultrastructural Investigation

The fixative samples were washed with physiological saline or 0.1 M phosphate buffer at pH 7.2. Then, the pellets were put into 1% osmium tetraoxide (OSO_4_) for 1-2 h at 4°C and rinsed in the buffer for 2 min. The samples were dehydrated in a series of acetone concentrations (25, 50, 75, and 100%) for 5 min. The tissues were infiltrated using propylene oxide. Epon/araldite was used to embed the specimen for 48 h under heating. Capsulated samples were sectioned by using the Ultratome machine at 20–30 nm thickness. The sections were collected on metal mesh grids and stained with toluidine blue for orientation. The grids were stained with 4% uranyl acetate for 5 min and then rinsed in a series of four beakers of pure water. After rinsing, the grids were stained with 1% lead acetate for 5 min, rinsed again in water, and stored in a grid box until observed [39].

Prepared grids were visualized by transmission electron microscopy (TEM) (JOEL 1400 Plus, Japan) for interpretation of cell changes. A combination of bright-field imaging was done as described above.

### 2.7. Statistical Analysis

The values of cell viability were estimated by using the Excel Software programme (Microsoft 2000). All data presented as mean ± SE were subjected to analysis of variance (ANOVA), and means were compared to significance by Student–Newman–Keuls at the probability of 0.05 [40].

## 3. Results

**Figure 1:**
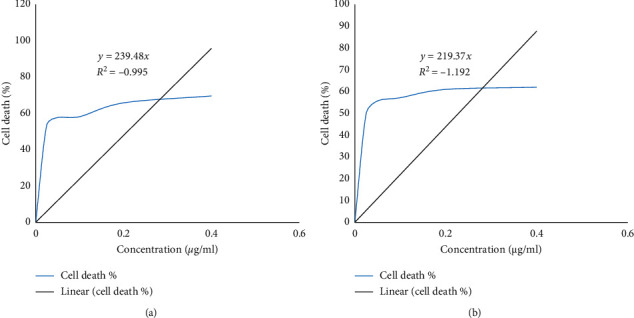
Acute toxic effect (cell death %) of the examined pesticides: (a) imidacloprid and (b) glyphosate on WPM-Y.1 cell line after 24 h exposure estimated as IC_50_.

**Figure 2:**
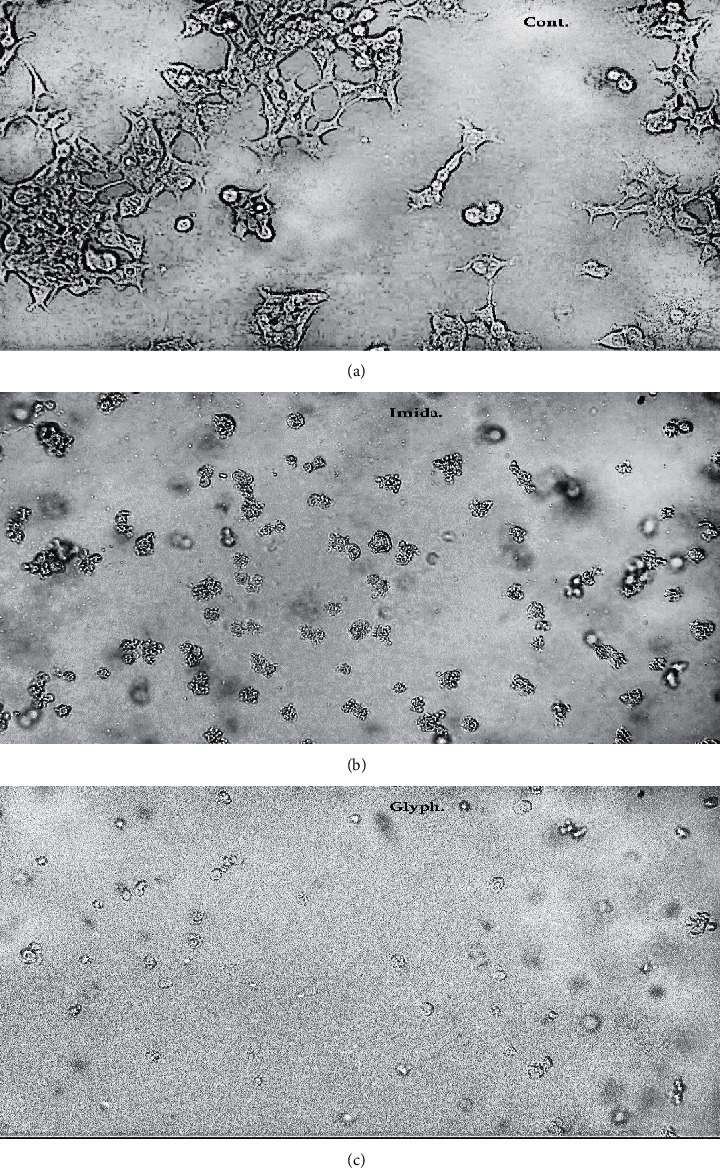
Pesticides-induced morphological changes in WPM-Y.1 cells. WPM-Y.1 cells were seeded in 12-well plates and allowed to grow for 20 h. Thereafter, cells were exposed to the IC_50_ level, 0.023 and 0.025 mM of imidacloprid and glyphosate, respectively, for 24 h. Significant alterations were induced in cell membranes and destructed their cytoplasm and microvilli which appeared as desorbed cells. The morphological changes were analyzed using the LABOMED inverted microscope, USA. Images selected were performed with a magnification of 20x.

WPM-Y.1 cells in these experiments showed growth rates around 24 h in the control medium. Treatments of imidacloprid and glyphosate induced rapid decrease in cell viability depending on their physicochemical properties when compared with control cells (Figure 1). The median inhibition concentration (IC_50_) values were 0.023 (*y* = 239.4*x*; *R*^2^ = − 0.995) and 0.025 mM (*y* = 219.37*x*; *R*^2^ = − 1.192) for imidacloprid and glyphosate, respectively. The endpoints of their cytotoxicity were confirmed by the defects which were induced in the exposed cells within 24 h compared with the untreated cells (Figure 2). Significant alterations were induced in the cell membrane and destructed their cytoplasm. The treated cells appeared in absorbed and destructed forms with few numbers than untreated cells. The coupling of microscopic investigation with the colorimetric assay of cell viability provides a good signature for the potential cytotoxic effects of the examined pesticides.

### 3.1. LDH

**Figure 3:**
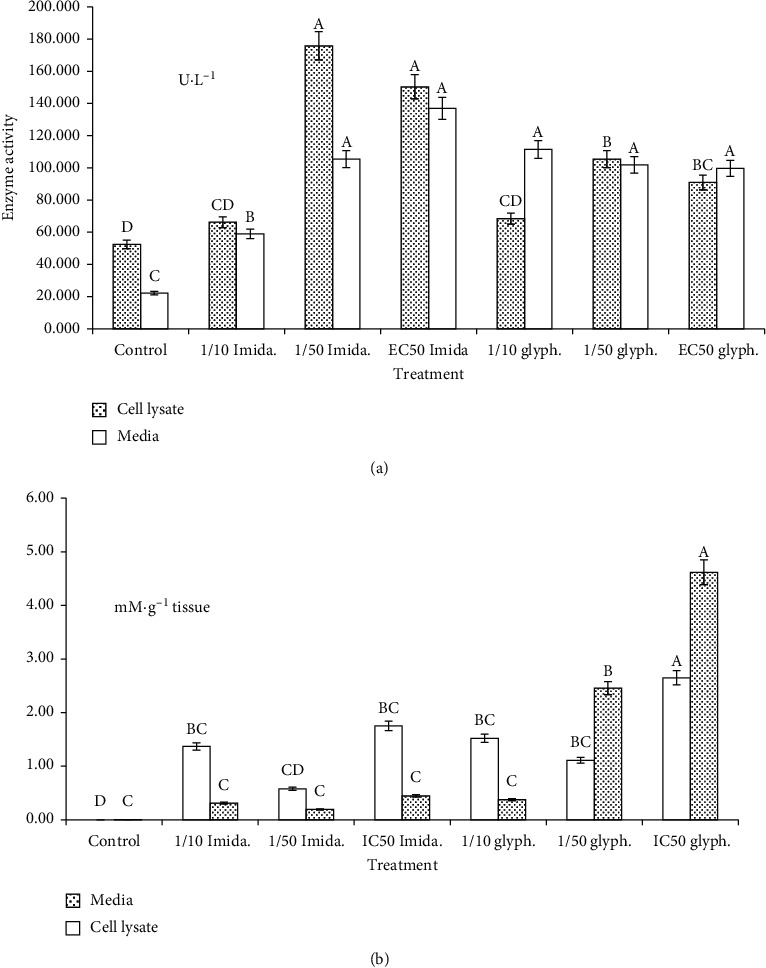
(a) Lactate dehydrogenase (LDH) activity (U L^−1^) and (b) malondialdehyde (MDA) level (mM g^−1^ tissue) in WPM-Y.1 cells exposed to different concentrations (mM) of imidacloprid and glyphosate. Each value indicates the mean of 3 replicates ± SE.

Sublethal concentrations of the examined pesticides significantly induced increase in the enzyme activity in both media and cell lysate (*P* < 0.05) (Figure 3(a)). The activity in cell lysate homogenate was greater than in media compared with untreated cells for the all treatments. Treatments of imidacloprid induced increase in enzyme activity greater than those of glyphosate. In cell lysate homogenate, the activity was as follows: 150.38, 66.23, 175.82 U·L^−1^ and 90.92, 68.43, and 105.38 U·L^−1^ for IC_50_, 1/10 IC_50_, and 1/50 IC_50_ treatments of imidacloprid and glyphosate, respectively. Regarding media of the cells, the order was 137.01, 58.91, and 105.43 U·L^−1^ and 99.72, 111.41, and 101.83 U·L^−1^ for the same manner as described above.

### 3.2. MDA

All treatments significantly increased MDA levels in both media and cell lysate in comparison with the control group (*P* < 0.05) (Figure 3(b)). In the media of the cells, all treatments of imidacloprid did not exceed 0.45 m·Mg^−1^ tissue in the case of IC_50_ treatment. Regarding cell lysate, MDA levels displayed the order: 2.65, 1.52, and 1.11 m·Mg^−1^ tissue and 1.75, 1.37, and 0.58 m·Mg^−1^ tissue for IC_50_, 1/10 IC_50_, and 1/50 IC_50_ treatments, respectively. However, glyphosate increased MDA levels: 4.62, 0.37, and 2.46 m·Mg^−1^ tissue for the same manner as described above for imidacloprid.

### 3.3. Antioxidant Defense Enzymes

**Figure 4:**
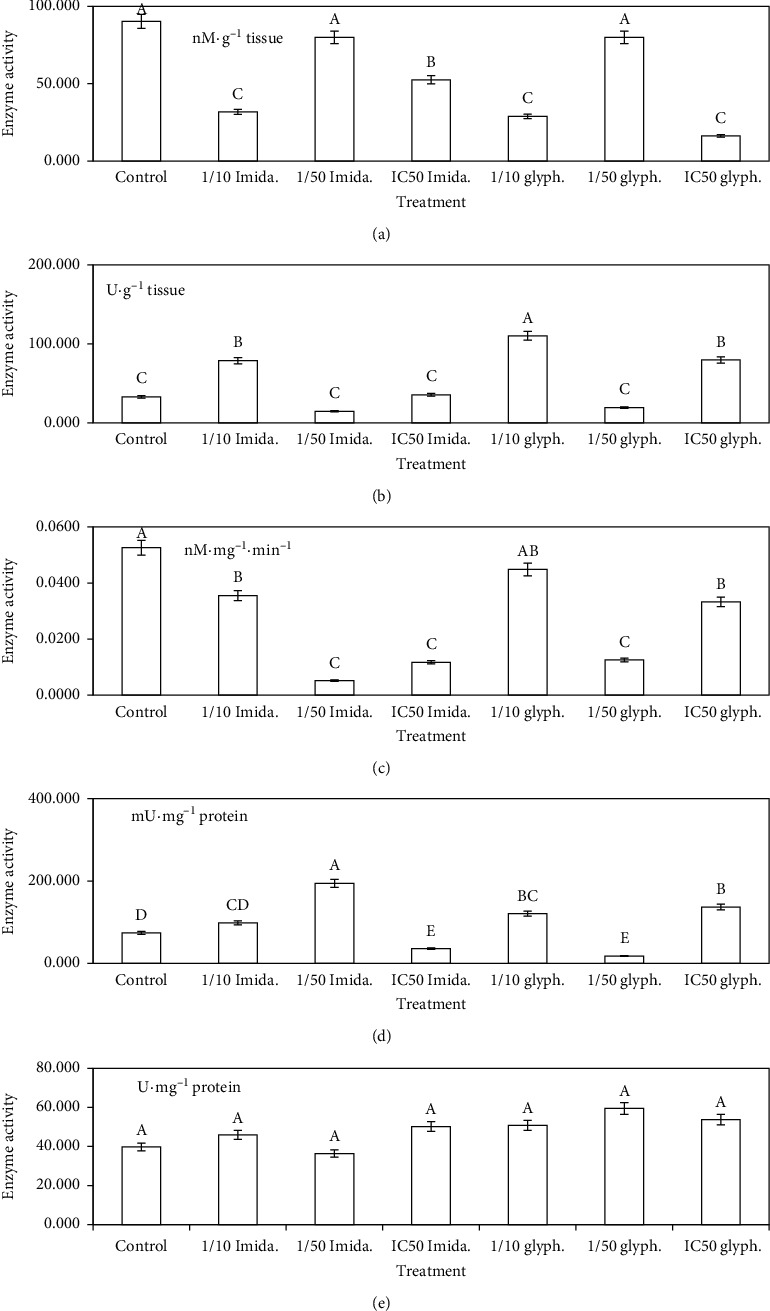
(a) Glutathione content (GSH), (b) catalase (CAT), (c) glutathione-S-transferase (GST), (d) glutathione peroxidase (GPx), and (e) glutathione reductase (GR) activities, respectively, in WPM-Y.1 cells exposed to different concentrations (mM) of imidacloprid and glyphosate. Each value represents the mean of 3 replicates ± SE. The same letters indicate no significant difference at the 0.05 level.

All treatments of the examined pesticides induced a rapid decrease in the GSH content in the homogenate of cell lysate (*P* < 0.05) (Figure 4(a)). In the case of imidacloprid, the decline in the GSH content was in the following order: 52.54, 31.82, and 79.92 n·Mg^−1^ tissue for IC_50_, 1/10, and 1/50 IC_50_ treatments, respectively. Glyphosate treatment at the IC_50_ level induced significant decline (17.94 n·Mg^−1^ tissue) compared with the control (90.28 n·Mg^−1^ tissue). Other treatments: 1/10 and 1/50 IC_50_ induced decrease in the GSH content with values: 28.86 and 79.92 n·Mg^−1^ tissue.

Two levels: IC_50_ and 1/10 IC_50_ of imidacloprid and glyphosate treatments were the only induced increase in the CAT activity, but 1/50 IC_50_ treatment exhibited significant decrease in the enzyme activity compared with the control group (*P* < 0.05) (Figure 4(b)). Regarding imidacloprid, 1/10 IC_50_ treatment exhibited the greatest activity (78.68 U·mg^−1^ protein), followed by IC_50_ treatment (35.65 U·mg^−1^ protein). The least activity (14.77 U·mg^−1^ protein) was recorded for 1/50 IC_50_ treatment. However, treatment (1/10 IC_50_ of glyphosate) exhibited the greatest activity (110.29 U·mg^−1^ protein), followed by IC_50_ treatment (79.67 U·mg^−1^ protein).

All treatments of the examined pesticides exhibited a significant decrease in the GST activity compared with the control group (*P* < 0.05) (Figure 4(c)). The least activity (0.0052 nM·mg^−1^ protein min^−1^) was recorded for IC_50_ treatment of imidacloprid, followed by IC_50_ treatment (0.0117 nM·mg^−1^ protein min^−1^). Regarding glyphosate, 1/10 IC_50_ treatment exhibited the greatest activity (0.0448 nM·mg^−1^ protein min^−1^), followed by IC_50_ and 1/50 IC_50_ treatments (0.0333 and 0.0125 nM·mg^−1^ protein min^−1^).

Regarding GPx activity, all treatments induced an increase in the enzyme activity, except IC_50_ treatment of imidacloprid and 1/50 IC_50_ treatment of glyphosate were the only induced significant decrease in the enzyme activity (*P* < 0.05) (Figure 4(d)). Treatment, 1/50 IC_50_ of imidacloprid, induced the greatest activity (194.62 n·Mg^−1^ tissue), followed by 1/10 IC_50_ treatments of both glyphosate or imidacloprid (120.86 and 98.26 n·Mg^−1^ tissue), respectively. However, 1/50 IC_50_ treatment of glyphosate exhibited the least activity (17.94 n·Mg^−1^ tissue) compared with the control group which did not exceed 74.27 n·Mg^−1^ tissue.

All treatments induced a slight increase in the GR activity compared with the control group (*P* < 0.05) (Figure 4(e)). Imidacloprid treatments induced activity in the following order: 50.18, 45.98, and 36.39 U·mg^−1^ tissue for IC_50_, 1/10 IC_50_, and 1/50 IC_50_ treatments, respectively. In case of glyphosate, the activity was in the following order: 53.80, 50.85, and 59.46 U·mg^−1^ tissue, in the same manner as described above.

### 3.4. Histopathological Defects of the Cells

**Figure 5:**
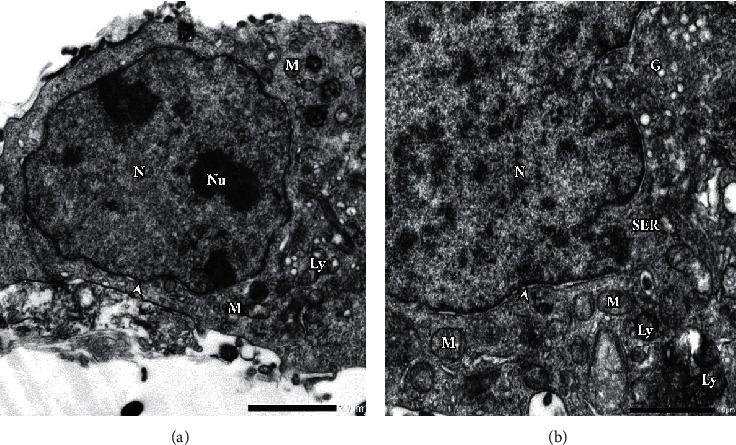
(a) Electron micrograph illustrates the section of untreated WMP-Y. 1 cells with regular nuclear membrane (head arrow). Smooth endoplasmic reticulum (SER), golgi bodies (G), and mitochondria (M) with light dense cristae. Highly distributed lysosomes (Ly). Moreover, significant double nuclei (Nu) were observed (F_4_G_1_-OsO_4_ fixed-uranyl acetate lead citrate stained preparation, 4000x). (b) High magnification showing normal distribution of chromatin and normal distribution of spherical mitochondrial organelle around the nucleus. Highly and distribution of golgi bodies and some vacuoles (8000x).

The sections of the harvested cells under TEM observation showed significant defects after pesticide treatments at the 1/10 IC_50_ level. In control (untreated) cells, it was observed a regular nuclear membrane and smooth endoplasmic reticulum (SER) (Figure 5). Also, a normal distribution of mitochondrial (M) organelle with light dense of cristae around the nucleus (N) was observed. There were highly dense Golgi bodies (G) distributed in the cytoplasm, and some vacuoles (V) were noted.

**Figure 6:**
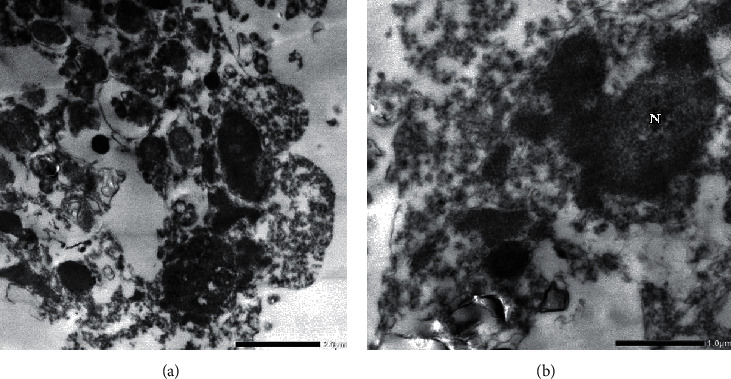
(a) Electron micrograph of imidacloprid-treated cells (0.0023 mM) illustrates destructed cell's organelles and no cellular membrane appeared (F_4_G_1_-OsO_4_ fixed-uranyl acetate lead citrate stained preparation, 4000x). (b) High magnification at 8000x shows nuclear-destructed components.

**Figure 7:**
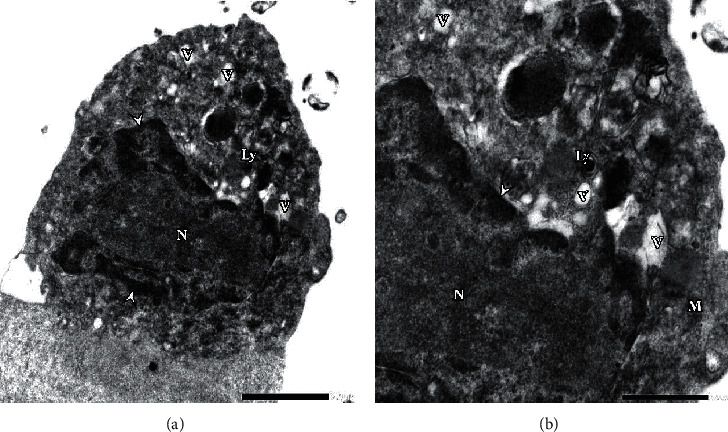
(a) Electron micrograph of glyphosate-treated cells (0.0025 mM) illustrates irregular nuclear membrane with highly migrated chromatin (head arrow) and lack of nuclei (Nu). Rough endoplasmic reticulum (RER), high distributed vacuoles (V), and some autolysomes (Ly) were noted (F_4_G_1_-OsO_4_ fixed-uranyl acetate lead citrate stained preparation, 4000x). (b) High magnification at 8000x shows compacted organelles and a lack of nuclei.

Regarding imidacloprid-treated cells, destructed organelles of the cells and significant defects in their components were noted (Figure 6). Moreover, no cellular membrane appeared, and there were destructed components of the nucleus (N). In glyphosate-treated cells, nucleus (N) with irregular nuclear membrane and high-migrated chromatin and lack of nuclei (Nu) was observed (Figure 7). Also, increase in vacuoles (V) and rough endoplasmic reticulum (RER) appeared. Some lysosomes (Ly) showed an autophagy (phagolysosomes) pattern. In general observation, compacted cell organelles and significant lack of mitochondria (M) were noted.

## 4. Discussion

We demonstrate the cytotoxicity of two pesticides which are extensively used in the agricultural sector of Egypt. Also, they were examined under low, environmentally, and physiologically relevant concentrations that may be found in the groundwater systems surrounding the agricultural application of the crops. The effects of the examined pesticides obtained in this work do not closely seem to be related to their mode of action in insects or weeds control. Responses of the tested human cells explored in this study to the potential toxic effect of imidacloprid and glyphosate differed markedly. WPM-Y.1 cell line is widely used as a cytotoxic model to examine these pesticides at concentrations that may receive human and other mammalian cellular systems. In fact, the use of cell lines along with biomonitoring data could permit a proper understanding of environmental metal/chemical toxicity, holistically [41]. Several toxicological test animal systems are undependable, as the exemplary mouse, rabbit, and guinea pig systems are not a true representative of the human body. Therefore, there are large gaps in the mirror of toxicity models with animals and humans [42]. Nevertheless, all popular animal models used in toxicology are mammals. Furthermore, the requirement of a high number of animals in toxicity studies is so much, for numerous test chemicals like 30.000 or more in number, which discourages the use of whole animals in assay systems [43]. Therefore, the use of cell lines in toxicology had been well recognized [44].

The present findings employed short exposure (24 h), but the previous demonstration showed a time amplifying effect, where the differential toxicity between the glyphosate and its formulation (Roundup^®^) was increased by 5 times in 72 h [45]. It appears that, with cell lines and short exposures, we underestimate by far the direct toxicity of the products in the long term. In the in vivo case, the metabolism may reduce the toxic effects but can be compensated or amplified by bioaccumulation and/or the combined effect of the pesticide and the adjuvants.

The present data indicate that imidacloprid and glyphosate caused cytotoxic effects on human normal cells and may be effective in vivo during long-term exposure with low doses for humans. Mechanism of pesticides inside the cell is intracellular reactive oxygen species (ROS) and disorganization of the cells [46]. As documented in the literature, toxicants increase ROS levels inside the cells which damage different cell organelles and promote apoptosis [47]. Oxidative stress is also known to disturb cellular membranes by altering tight junction molecules [29, 30]. As stated by Ilboudo et al. [48], pesticides: deltamethrin, fenitrothion, fipronil, lambda-cyhalothrin, and teflubenzuron induced functional alterations of the epithelial Caco-2 cell layer in correlation with the pro-oxidative activity of these chemicals.

The present results are in accordance with that observed in the cytotoxicity of other compounds in vitro models. For example, cytotoxicity of deltamethrin was indeed observed in cortical neurons [49], in rat spermatozoa [50], and in SH-SY5Y cells [51]. According to Feng et al. [17], high concentrations of imidacloprid led to some toxic and genotoxic effects in human peripheral lymphocytes. Also, imidacloprid is able to inhibit the growth of flounder gill (FG) cell culture causing severe injury to the mitochondria. It is considered as a probabe target of imidacloprid [52]. In in vitro studies, Yao et al. [53] have indicated that acetamiprid may induce ROS generation in bacteria. However, the incubation of imidacloprid with Yurkat cells and lymphocytes did not increase the production of ROS [19]. Also, the obtained results are in accordance with that obtained by Valko et al. [54], where neonicotinoid insecticides were acted as a source of ROS or free radicals in the treated human cells. On the contrary, myclobutanil at different concentrations reduced the cell viability. However, the cytotoxic effect was partial in low concentrations (1–10 *μ*g·ml^−1^), but in high concentrations, there was near-total cell death [55].

The increased MDA levels in the cells explain the success of LPO process and the failure of antioxidant defense mechanisms to prevent the formation of excessive free radicals [56]. LDH is one of the cellular death biomarkers that can be measured in many cases, where its release in the media of cultured cells is associated with cell and membrane damage [57, 58]. In the present study, the coupling increase in MDA and LDH in cell lysate and media is considered a good biomarker for the cytotoxic effect of imidacloprid and glyphosate in the tested cells. Recently, the potential toxic effect of the examined pesticides on human prostate epithelial WPM-Y.1 cell line is associated with oxidative stress and cell damage induction, whereas, during apoptosis, the potential difference in mitochondrial membrane is lost and some proteins such as cytochrome C are released into the cytosol. When mitochondria are damaged, the antioxidant system is compromised resulting in an increased production of ROS and high induction of oxidative stress [59]. The present findings showed that the examined pesticides induced dose-dependent cytotoxicity characterized by cell death attributing to apoptotic and necrosis mechanisms, alterations of oxidative stress enzymes activity, increased levels of LPO, and LDH activity as well as histopathological alterations. The sum of such biomarkers concerning cytotoxicity of imidacloprid and glyphosate displays true results in this issue without any contrast in this fact.

## 5. Summary and Conclusion

Our study is the first investigation that obtains the potential cytotoxic actions of imidacloprid and glyphosate in epithelial human prostatic WPM-Y.1 cell line in vitro. At low concentrations (mM), the examined pesticides significantly reduced cell viability and caused cell death. Coupling of cell viability, oxidative stress, and histopathological alterations provides good tools to assess the cytotoxicity of pesticides in vitro at low concentrations. Moreover, the abnormal damage of cell structure is considered an important signal of organ dysfunction.

## Data Availability

The data underlying the findings of the manuscript are publicly available.

## References

[1] NPIC (2010). *Imidacloprid: Technical Fact Sheet*.

[2] Morrissey C. A., Mineau P., Devries J. H. (2015). Neonicotinoid contamination of global surface waters and associated risk to aquatic invertebrates: a review. *Environment International*.

[3] Myers J. P., Antoniou M. N., Blumberg B. (2016). Concerns over use of glyphosate-based herbicides and risks associated with exposure: a consensus statement. *Environmental Health*.

[4] Arias A. H., Buzzi N. S., Pereira M. T., Marcovecchio J. E., Stoytcheva M. (2011). Pesticides reaching the environment as a consequence of inappropriate agricultural practices in Argentina. *Pesticides-Formulations, Effects, Fate*.

[5] Salako A. A., Sholeye O. O., Dairo O. O. (2012). Beyond pest control: a closer look at the health implication of pesticides usage. *Journal of Toxicology and Environmental Health Sciences*.

[6] Tomizawa M., Casida J. E. (2005). Neonicotinoid insecticide toxicology: mechanisms of selective action. *Annual Review of Pharmacology and Toxicology*.

[7] Simon-Delso N., Amaral-Rogers V., Belzunces L. P. (2015). Systemic insecticides (neonicotinoids and fipronil): trends, uses, mode of action and metabolites. *Environmental Science and Pollution Research International*.

[8] Ihara M., Shimomura M., Ishida C. (2007). A hypothesis to account for the selective and diverse actions of neonicotinoid insecticides at their molecular targets, nicotinic acetylcholine receptors: catch and release in hydrogen bond networks. *Invertebrate Neuroscience*.

[9] Millar N. S., Denholm I. (2007). Nicotinic acetylcholine receptors: targets for commercially important insecticides. *Invertebrate Neuroscience*.

[10] EPA (2003). *Clothianidin; Pesticide Tolerances-Federal Register*.

[11] WHO (2009). *The WHP Recommended Classification of Pesticides by Hazard and Guidelines to Classification*.

[12] Fishel F. M. (2010). *Pesticide Toxicity Profile: Neonicotinoid Pesticides*.

[13] NRC (1989). *Biologic Markers in Reproductive Toxicology*.

[14] Caron-Beaudoin É., Denison M. S., Sanderson J. T. (2016). Effects of neonicotinoids on promoter-specific expression and activity of aromatase (CYP19) in human adrenocortical carcinoma (H295R) and primary umbilical vein endothelial (HUVEC) cells. *Toxicological Sciences*.

[15] Zang Y., Zhong Y., Luo Y., Kong Z. M. (2000). Genotoxicity of two novel pesticides for the earthworm, *Eisenia fetida*. *Environmental Pollution*.

[16] Feng S., Kong Z., Wang X., Zhao L., Peng P. (2004). Acute toxicity and genotoxicity of two novel pesticides on amphibian, *Rana N. Hallowell*. *Chemosphere*.

[17] Feng S., Kong Z., Wang X., Peng P., Zeng E. Y. (2005). Assessing the genotoxicity of imidacloprid and RH-5849 in human peripheral blood lymphocytes in vitro with comet assay and cytogenetic tests. *Ecotoxicology and Environmental Safety*.

[18] Calderón-Segura M. E., Gómez-Arroyo S., Villalobos-Pietrini R. (2012). Evaluation of genotoxic and cytotoxic effects in human peripheral blood lymphocytes exposed *In vitro* to neonicotinoid insecticides news. *Journal of Toxicology*.

[19] Costa C., Silvari V., Melchini A. (2009). Genotoxicity of imidacloprid in relation to metabolic activation and composition of the commercial product. *Mutation Research/Genetic Toxicology and Environmental Mutagenesis*.

[20] Cox C. (1998). Glyphosate (Roundup^®^). *Journal Pesticide Reform*.

[21] Kwiatkowska M., Jarosiewicz P., Bukowska B. (2013). Glyphosate and its formulations--toxicity, occupational and environmental exposure (Polosh). *Medycyna Pracy*.

[22] Kwiatkowska M., Jarosiewicz P., Michałowicz J., Koter-Michalak M., Huras B., Bukowska B. (2016). The impact of glyphosate, its metabolites and impurities on viability, ATP level and morphological changes in human peripheral blood mononuclear cells. *PLoS One*.

[23] Gasnier C., Dumont C., Benachour N., Clair E., Chagnon M.-C., Séralini G.-E. (2009). Glyphosate-based herbicides are toxic and endocrine disruptors in human cell lines. *Toxicology*.

[24] Mañas F., Peralta L., Raviolo J. (2009). Genotoxicity of glyphosate assessed by the comet assay and cytogenetic tests. *Environmental Toxicology and Pharmacology*.

[25] Li Q., Lambrechts M. J., Zhang Q. (2013). Glyphosate and AMPA inhibit cancer cell growth through inhibiting intracellular glycine synthesis. *Drug Design, Development and Therapy*.

[26] IARC (2015). Glyphosate. *Some Organophosphate Insecticides and Herbicides: Diazinon, Glyphosate, Malathion, Parathion and Tetrachlorvinophos*.

[27] Ishiyama M., Tominaga H., Shiga M., Sasamoto K., Ohkura Y., Ueno K. (1996). A combined assay of cell viability and in vitro cytotoxicity with a highly water-soluble tetrazolium salt, neutral red and crystal violet. *Biological & Pharmaceutical Bulletin*.

[28] Tebourbi O., Sakly M., Rhouma K. B. (2011). Molecular mechanisms of pesticide toxicity. *Pesticides in the Modern World-Pests Control and Pesticides Exposure and Toxicity Assessment*.

[29] Lee H.-S., Namkoong K., Kim D.-H. (2004). Hydrogen peroxide-induced alterations of tight junction proteins in bovine brain microvascular endothelial cells. *Microvascular Research*.

[30] Hashimoto K., Oshima T., Tomita T. (2008). Oxidative stress induces gastric epithelial permeability through claudin-3. *Biochemical and Biophysical Research Communications*.

[31] Mc Queen M. J. (1975). True Arrhenius relationships of human lactate dehydrogenase. *Z Klin Chem Klin Biochem*.

[32] Rice-Evans C. A., Diplock A. T., Symons M. C. R. (1991). *Technique in Free Radical Research*.

[33] Beulter E., Duron O., Kelly B. M. (1963). Improved method for the determination of blood glutathione. *Journal of Laboratory and Clinical Medicine*.

[34] Beers R. F., Sizer I. W. (1952). Spectrophotometric method for measuring the breakdown of hydrogen peroxide by catalase. *Journal of Biological Chemistry*.

[35] Habig W. H., Jakoby W. B. (1981). [27] Glutathione S-transferases (rat and human). *Methods in Enzymology*.

[36] Flohe L., Gunzler W. A. (1984). Assays of glutathione peroxidase. *Methods of Enzymology*.

[37] Goldberg D. M., Spooner R. J., Bregmay H. V. (1983). Glutathione reductase. *Methods of Enzymatic Analysis*.

[38] Lowry O. H., Rosebrough N. J., Farr A. L., Randall R. J. (1951). Protein measurement with the folin phenol reagent. *The Journal of Biological Chemistry*.

[39] Reynold E. S. (1963). The use of lead citrate of high pH as an electron-opaque stain in electron microscopy. *The Journal of Cell Biology*.

[40] Cohort Software Ltd. (1985). *Costal User Manual, Version 3*.

[41] Nakadai A., Li Q., Kawada T. (2008). Chlorpyrifos induces apoptosis in human mono-cyte cell line U937. *Toxicology*.

[42] Tzimas G., Thiel R., Chahoud I., Nau H. (1997). The area under the concentration-time curve of all-trans-retinoic acid is the most suitable pharmacokinetic correlate to the embryotoxicity of this retinoid in the rat. *Toxicology and Applied Pharmacology*.

[43] Gillbert P. M., Havenstrite K. L., Magnusson K. E. G. (2010). Substrate elasticity regulates skeletal muscle stem cell self-renewal in culture. *Science*.

[44] Rosler E. S., Fisk G. J., Ares X. (2004). Long-term culture of human embryonic stem cells in feeder-free conditions. *Developmental Dynamics*.

[45] Benachour N., Sipahutar H., Moslemi S., Gasnier C., Travert C., Séralini G. E. (2007). Time- and dose-dependent effects of roundup on human embryonic and placental cells. *Archives of Environmental Contamination and Toxicology*.

[46] Matés J. M., Segura J. A., Alonso F. J., Márquez J. (2010). Roles of dioxins and heavy metals in cancer and neurological diseases using ROS-mediated mechanisms. *Free Radical Biology and Medicine*.

[47] Valencia A., Kochevar I. E. (2006). Ultraviolet A induces apoptosis via reactive oxygen species in a model for Smith-Lemli-Opitz syndrome. *Free Radical Biology and Medicine*.

[48] Ilboudo S., Fouche E., Rizzati V., Toé A. M., Gamet-Payrastre L., Guissou P. I. (2014). In vitro impact of five pesticides alone or in combination on human intestinal cell line Caco-2. *Toxicology Reports*.

[49] Wu A., Li L., Liu Y. (2003). Deltamethrin induces apoptotic cell death in cultured cerebral cortical neurons. *Toxicology and Applied Pharmacology*.

[50] Abdallah F. B., Hamden K., Galeraud-Denis I., El Feki A., Keskes-Ammar L. (2010). An in vitro study on reproductive toxicology of deltamethrin on rat spermatozoa. *Andrologia*.

[51] Romero A., Ramos E., Castellano V. (2012). Cytotoxicity induced by deltamethrin and its metabolites in SH-SY5Y cells can be differentially prevented by selected antioxidants. *Toxicology in Vitro*.

[52] Su F., Zhang S., Li H., Guo H. (2007). *In vitro* acute cytotoxicity of neonicotinoid insecticide imidacloprid to gill cell line of flounder *Paralichthy olivaceus*. *Chinese Journal of Oceanology and Limnology*.

[53] Yao X. H., Min H., Lv Z. M. (2006). Response of superoxide dismutase, catalase and ATPase activity in bacteria exposed to acetamiprid. *Biomedical and Environmental Sciences*.

[54] Valko M., Rhodes C. J., Moncol J., Izakovic M., Mazur M. (2006). Free radicals, metals and antioxidants in oxidative stress-induced cancer. *Chemico-Biological Interactions*.

[55] Stellavato A., Lamberti M., Pirozzi A. V. A., de Novellis F., Schiraldi C. (2006). Myclobutanil worsens nonalcoholic fatty liver disease: an in vitro study of toxicity and apoptosis on HePG2 cells. *Toxicology Letters*.

[56] Kim H.-Y., Kim J.-K., Choi J.-H. (2010). Hepatoprotective effect of pinoresinol on carbon tetrachloride-induced hepatic damage in mice. *Journal of Pharmacological Sciences*.

[57] Osman K. A. (1999). Lindane, chlorpyrifos and paraquat induced oxidative stress in female rats. *Alexandria Journal Agricultural Research*.

[58] Bakry F. A., Hasheesh W. S., Hamdi S. A. H. (2011). Biological, biochemical, and molecular parameters of *Helisoma duryi* snails exposed to the pesticides malathion and deltamethrin. *Pesticide Biochemistry and Physiology*.

[59] Pope S., Land J. M., Heales S. J. R. (2008). Oxidative stress and mitochondrial dysfunction in neurodegeneration; cardiolipin a critical target?. *Biochimica et Biophysica Acta*.

